# Targeting PMN‐MDSCs via CD300ld receptor for cancer immunotherapy

**DOI:** 10.1002/ctm2.1534

**Published:** 2024-01-11

**Authors:** Yuwei Li, Chaoxiong Wang, Zhigang Lu, Min Luo

**Affiliations:** ^1^ Institute of Pediatrics of Children's Hospital of Fudan University Shanghai Key Laboratory of Medical Epigenetics Institutes of Biomedical Sciences Fudan University Shanghai China; ^2^ The Fifth People's Hospital of Shanghai Institutes of Biomedical Sciences Fudan University Shanghai China; ^3^ State Key Laboratory of Cell Biology Center for Excellence in Molecular Cell Science Shanghai Institute of Biochemistry and Cell Biology University of Chinese Academy of Sciences Chinese Academy of Sciences Shanghai China

Immune checkpoint blockage has considerably improved patient survival but efficacy remains limited in many cases.^1^ The immune suppressive tumour microenvironment (TME), characterised by high content of myeloid cells, represents a major obstacle against multiple immunotherapies.^2^, ^3^ In recent years, sustained focus has been paid to neutrophils—a major part of myeloid cells within the milieu of cancer patients. These pathologically activated neutrophils exhibit potent immune suppressive activity and are functionally named polymorphonuclear myeloid‐derived suppressor cells (PMN‐MDSCs), emerging as a critical component in TME. PMN‐MDSCs are expanded in most types of cancer, which contribute to tumour progression and immune suppression, causing poor response to immunotherapy.^3^, ^4^ Here, we will concisely discuss the significance and inherent mechanisms of PMN‐MDSCs in tumourigenesis and resistance to therapy, along with recent advancements for therapeutic manipulation of these cells.

## 1 SIGNIFICANCE AND INHERENT MECHANISMS OF PMN‐MDSC

Neutrophils are polymorphonuclear granulocytes of the innate immune system that play key roles in defense against acute infection or inflammation. In cancer or some other chronic inflammation, the massive exposure of myeloid progenitors to tumour‐secreted growth factors and inflammatory mediators triggers aberrant granulopoiesis, significantly increases neutrophil release from bone marrow. These neutrophils, particularly in late‐stage cancers, are functionally perturbed, showing a high level of plasticity and potent immune suppressive activity, and thus often referred to as PMN‐MDSC.^4^, ^5^ PMN‐MDSCs and classical neutrophils are functionally different, however, few archetypal genetic and phenotypic markers exist to distinguish them in human and notably in mice. Recent discoveries of novel molecular markers, including CD84, LOX1, FATP2 and CD14, may help to separate immunosuppressive PMN‐MDSCs from classical neutrophils in a context‐dependent manner.^3^, ^4^

PMN‐MDSCs employ diverse mechanisms to promote tumour progression and limit anti‐cancer immune responses.^3^, ^5^ Immune suppression is the fundamental property of PMN‐MDSCs. PMN‐MDSCs express a multitude of mediators, such as reactive oxygen species (ROS), inducible nitric oxide synthase, arginine decarboxylase 1, prostaglandins and ligands of immune checkpoint molecules, causing T‐cell dysfunction and derangement of T‐cell‐driven anti‐tumour immunity. Other situation has also been described that MDSC‐enabled pathways induce regulatory T‐cell expansion. Beyond impacting immune regulation, ROS released by PMN‐MDSCs contributes to DNA damage and genetic instability in cancer cells. Notably, PMN‐MDSCs can produce various pro‐angiogenic factors that stimulate tumour angiogenesis, such as Bv8, matrix metalloproteinase 9 and vascular endothelial growth factor A. Moreover, neutrophil extracellular traps released under conditions such as hypoxia, complement or fatty acid stimulation can induce dormant cancer cell division and trap circulating tumour cells to promote formation of premetastatic niche.^6^, ^7^

Growing evidence underscores the strong correlation between high frequencies of circulating and tumour‐infiltrating neutrophils and unfavourable prognosis in cancer patients. Numerous clinical investigations have delineated that elevated neutrophil counts and neutrophil:lymphocyte ratio (NLR) in peripheral blood are predictive of poor outcomes for individuals affected by various malignancies.^8^ This prognostic significance of NLR was strengthened via a comprehensive meta‐analysis encompassing 100 trials featuring 40 559 patients and 22 distinct types of solid tumours.^9^ In addition, based on gene expression profiling data of tumours from more than 18 000 patients, the CIBERSORT project revealed that tumour‐associated neutrophils emerge as the single‐cell population most highly associated with mortality across broad categories of cancers.^10^ Neutrophils also profoundly affect intrinsic responses to diverse anticancer therapeutic strategies. Elevated neutrophil infiltration is linked to diminished responsiveness towards chemotherapy and radiotherapy.^11^, ^12^ Meanwhile, peripheral blood neutrophilia and high NLR positively correlate with suboptimal responsiveness to immune checkpoint inhibitors.^5^ Tissue assessments of non‐small cell lung cancer patients indicated a subset of individuals with high numbers of PMN‐MDSCs were refractory to Immune Checkpoint Inhibitors (ICI) therapy, and the CD8^+^‐T‐cell:neutrophil ratio within the TME effectively distinguished responders from non‐responders.^13^

## 2 TARGETING PMN‐MDSCS FOR CANCER IMMUNOTHERAPY

Targeting PMN‐MDSCs has emerged as an attractive strategy for cancer treatment, focusing on reducing their recruitment, inhibitory effects and direct depletion. Several drugs that target PMN‐MDSCs have demonstrated superior anti‐tumour properties when paired with checkpoint inhibitors. The administration of chemokine receptor CXCR1/2 inhibitor, SX‐682, reduce tumour growth by blocking PMN‐MDSC recruitment and show synergistic effects with anti‐PD1 antibodies.^14^ The inhibition of FATP2 abrogated the suppressive functions of PMN‐MDSCs and sensitised anti‐CTLA4 antibody treatment.^15^ Activation of DR5 through agonistic antibodies induces apoptosis of PMN‐MDSCs, potentiating the efficacy of anti‐CTLA4 antibody treatment against tumours.^16^ Of note, concerns about these targeting include off‐target side‐effects and the compensatory myelopoiesis as a result of physiological homeostatic regulation.^3^, ^17^ Ongoing research is imperative to identify more specific and potent therapeutic targets.

In our recent study,^18^ we focused on the surface membrane proteins highly expressed by myeloid cells, and performed an in vivo Crispr‐Cas9 screen in a tumour‐bearing mouse model to identify key receptor(s) that is required for PMN‐MDSC recruitment and functionality. CD300ld was identified as a top candidate for tumour favouring receptor. CD300ld is a single‐pass transmembrane protein and belongs to CD300 family. We found that CD300ld expression is restricted to myeloid lineages, being high in neutrophils and low‐to‐non‐existent in other myeloid and lymphoid cells. Upon tumour bearing, CD300ld expression is up‐regulated in PMN‐MDSCs. CD300ld thus serves as a marker for PMN‐MDSCs/neutrophils. CD300ld knockout (KO) significantly impairs tumour development in multiple syngeneic and spontaneous tumour models in a PMN‐MDSC‐dependent manner. Single‐cell transcriptome analysis further revealed that loss of CD300ld significantly alters the pro‐ and anti‐tumour cell composition and the related immune pathways, remodelling the TME from immune suppressive to immune active. Given that the high levels of CD300ld expression are limited to PMN‐MDSCs, and these cells are substantially reduced in CD300ld KO mice tumours, the results further suggested the central roles of PMN‐MDSCs in the establishment of a tumour promoting microenvironment.

Mechanism investigation revealed that CD300ld is required for both the recruitment of PMN‐MDSCs to tumours and their suppression on T‐cell activity through STAT3–S100A8/A9 axis. Competitive blockade of CD300ld by its extracellular domain (ECD) inhibits the growth of established tumour in a way similar to CD300ld KO, and exhibits a significant anti‐tumour synergy effect with anti‐PD1, indicating that CD300ld can serve as a therapeutic target. Notably, no gross abnormalities were observed when treating mice with CD300ld ECD, and the CD300ld KO has little effect on mouse development, suggesting that targeting this molecule may be a safe therapeutic option.

**FIGURE 1:**
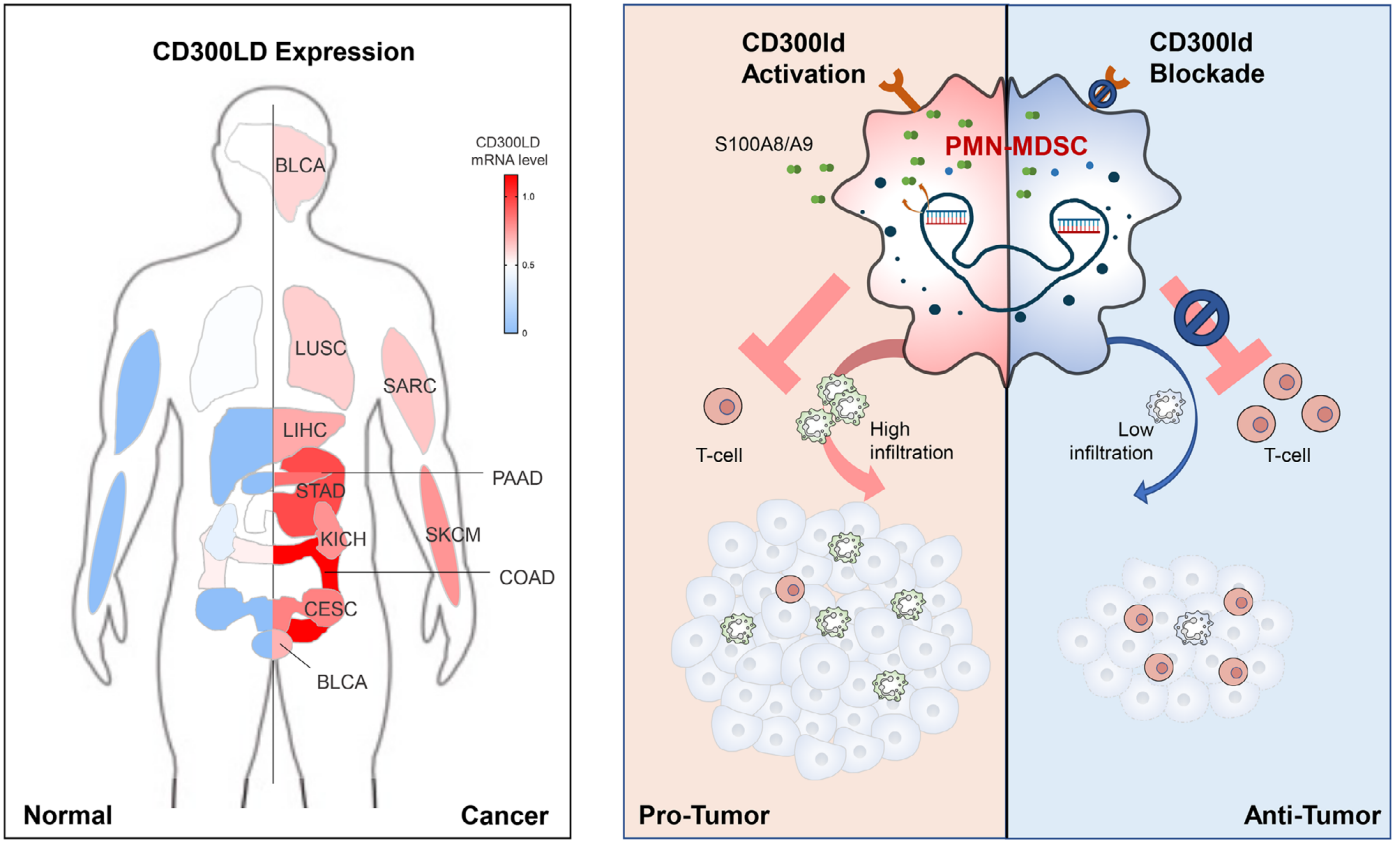
CD300ld expression and function in cancer. Left: increased expression of CD300ld in multiple types of human cancer. Raw data derived from The Cancer Genome Atlas Program (TCGA) database. Right: CD300ld acts as an immune suppressor on polymorphonuclear myeloid‐derived suppressor cells (PMN‐MDSCs) for tumour‐driven immune suppression.

Clinical relevance analysis showed that human CD300ld is up‐regulated in various tumour types (Figure 1), with a significant correlation between high CD300ld signalling and poor patient survival outcomes in melanoma patients. We also generated CD300ld humanised mice and demonstrated that the tumour promoting activity of Cd300ld is conserved between mouse and human.

In conclusion, this study has identified CD300ld as a specific surface marker and a critical immune suppressor on PMN‐MDSCs to promote tumour progression (Figure 1). Blockage of CD300ld remodels the immune‐suppressive TME and shows anti‐tumour efficacy, as well as synergistic effects with immune checkpoint inhibitors, providing a promising target for cancer immunotherapy and other systemic inflammatory disorders where PMN‐MDSCs are implicated.

## CONFLICT OF INTEREST STATEMENT

The authors declare they have no conflicts of interest.

## References

[1] Boussiotis VA . Molecular and biochemical aspects of the PD‐1 checkpoint pathway. N Engl J Med. 2016;375:1767‐1778. 27806234 10.1056/NEJMra1514296 PMC5575761

[2] Engblom C , Pfirschke C , Pittet MJ . The role of myeloid cells in cancer therapies. Nat Rev Cancer. 2016;16:447‐462. 27339708 10.1038/nrc.2016.54

[3] Veglia F , Sanseviero E , Gabrilovich DI . Myeloid‐derived suppressor cells in the era of increasing myeloid cell diversity. Nat Rev Immunol. 2021;21:485‐498. 33526920 10.1038/s41577-020-00490-y PMC7849958

[4] Quail DF , Amulic B , Aziz M , et al. Neutrophil phenotypes and functions in cancer: a consensus statement. J Exp Med. 2022;219:e20220011. 35522219 10.1084/jem.20220011 PMC9086501

[5] Jaillon S , Ponzetta A , Di Mitri D , et al. Neutrophil diversity and plasticity in tumour progression and therapy. Nat Rev Cancer. 2020;20:485‐503. 32694624 10.1038/s41568-020-0281-y

[6] Park J , Wysocki RW , Amoozgar Z , et al. Cancer cells induce metastasis‐supporting neutrophil extracellular DNA traps. Sci Transl Med. 2016;8:361ra138. 10.1126/scitranslmed.aag1711 PMC5550900 27798263

[7] Albrengues J , Shields MA , Ng D , et al. Neutrophil extracellular traps produced during inflammation awaken dormant cancer cells in mice. Science. 2018;361:eaao4227. 30262472 10.1126/science.aao4227 PMC6777850

[8] Shaul ME , Fridlender ZG . Tumour‐associated neutrophils in patients with cancer. Nat Rev Clin Oncol. 2019;16:601‐620. 31160735 10.1038/s41571-019-0222-4

[9] Templeton AJ , McNamara MG , Seruga B , et al. Prognostic role of neutrophil‐to‐lymphocyte ratio in solid tumors: a systematic review and meta‐analysis. J Natl Cancer Inst. 2014;106:dju124. 24875653 10.1093/jnci/dju124

[10] Gentles AJ , Newman AM , Liu CL , et al. The prognostic landscape of genes and infiltrating immune cells across human cancers. Nat Med. 2015;21:938‐945. 26193342 10.1038/nm.3909 PMC4852857

[11] Galdiero MR , Bianchi P , Grizzi F , et al. Occurrence and significance of tumor‐associated neutrophils in patients with colorectal cancer. Int J Cancer. 2016;139:446‐456. 26939802 10.1002/ijc.30076

[12] Posabella A , Kohn P , Lalos A , et al. High density of CD66b in primary high‐grade ovarian cancer independently predicts response to chemotherapy. J Cancer Res Clin Oncol. 2020;146:127‐136. 31853662 10.1007/s00432-019-03108-6 PMC11804757

[13] Kargl J , Zhu X , Zhang H , et al. Neutrophil content predicts lymphocyte depletion and anti‐PD1 treatment failure in NSCLC. JCI Insight. 2019;4:e130850. 31852845 10.1172/jci.insight.130850 PMC6975266

[14] Highfill SL , Cui Y , Giles AJ , et al. Disruption of CXCR2‐mediated MDSC tumor trafficking enhances anti‐PD1 efficacy. Sci Transl Med. 2014;6:237ra267. 10.1126/scitranslmed.3007974 PMC6980372 24848257

[15] Veglia F , Tyurin VA , Blasi M , et al. Fatty acid transport protein 2 reprograms neutrophils in cancer. Nature. 2019;569:73‐78. 30996346 10.1038/s41586-019-1118-2 PMC6557120

[16] Condamine T , Kumar V , Ramachandran IR , et al. ER stress regulates myeloid‐derived suppressor cell fate through TRAIL‐R‐mediated apoptosis. J Clin Invest. 2014;124:2626‐2639. 24789911 10.1172/JCI74056 PMC4038578

[17] Villanueva MT . Targeting cancer‐associated neutrophils. Nat Rev Drug Discov. 2019;18:419. 31160765 10.1038/d41573-019-00078-9

[18] Wang C , Zheng X , Zhang J , et al. CD300ld on neutrophils is required for tumour‐driven immune suppression. Nature. 2023;621:830‐839. 37674079 10.1038/s41586-023-06511-9

